# Epidemiology and disease burden of tuberculosis in south of Tunisia over a 22-year period: Current trends and future projections

**DOI:** 10.1371/journal.pone.0212853

**Published:** 2019-07-24

**Authors:** Houda Ben Ayed, Makram Koubaa, Lamia Gargouri, Maissa Ben Jemaa, Maroua Trigui, Fatma Hammemi, Mariem Ben Hmida, Abdelmajid Mahfoudh, Neila Zalila, Aida Mustapha, Chokri Masmoudi, Chakib Marrakchi, Sourour Yaich, Feriel Messaadi, Ali Ayedi, Jamel Damak, Mounir Ben Jemaa

**Affiliations:** 1 Community Health and Epidemiology Department, Hedi Chaker University Hospital, University of Sfax, Sfax, Tunisia; 2 Extra-pulmonary Research Unity, Hedi Chaker University Hospital, Sfax, Tunisia; 3 Infectious Diseases Department, Hedi Chaker University Hospital, University of Sfax, Sfax, Tunisia; 4 Department of Pediatrics, Hedi Chaker University Hospital, Sfax, Tunisia; 5 Regional Primary Health Care Directory, Sfax, Tunisia; Department of Internal Medicine, Federal Teaching Hospital Abakaliki, Ebonyi State, NIGERIA

## Abstract

**Background:**

Tuberculosis (TB) is a public health problem worldwide. Characterizing its trends over time is a useful tool for decision-makers to assess the efficiency of TB control programs. We aimed to give an update on the current chronological trends of TB in Southern Tunisia from 1995 to 2016 and to estimate future trajectories of TB epidemic by 2030.

**Methods:**

We retrospectively collected data of all notified TB new cases by the Center of Tuberculosis Control between 1995 and 2016 in South of Tunisia. Joinpoint Regression Analysis was performed to analyze chronological trends and annual percentage changes (APC) were estimated.

**Results:**

In the past 22 years, a total of 2771 cases of TB were notified in Southern Tunisia. The annual incidence rate of TB was 13.91/100,000 population/year. There was a rise in all forms of TB incidence (APC = 1.63) and in extrapulmonary tuberculosis (EPTB) (APC = 2.04). The incidence of TB increased in children and adult females between 1995 and 2016 (APC = 4.48 and 2.37, respectively). The annual number of TB declined in urban districts between 2004 and 2016 (APC = -2.85). Lymph node TB cases increased (APC = 4.58), while annual number of urogenital TB decreased between 1995 and 2016 (APC = -3.38). Projected incidence rates would increase to 18.13 and 11.8/100,000 population in 2030 for global TB and EPTB, respectively.

**Conclusions:**

Our study highlighted a rise in all forms of TB and among high-risk groups, notably children, females and lymph node TB patients in the last two decades and up to the next one.

## Introduction

Tuberculosis (TB) is a global pandemic which poses a serious threat to public health throughout the world [1]. Despite effective treatment, TB is the leading cause of death from a single infectious agent, ranking above Human Immunodeficiency Virus (HIV), excluding HIV-TB co-infection [2]. TB is a bacterial infection caused by different types of mycobacterium, notably *Mycobacterium tuberculosis* and *Mycobacterium bovis*. It may occur at different anatomical sites of the human body, affecting lung parenchyma, called pulmonary tuberculosis (PTB) or other anatomical sites outside parenchyma, designated extra-pulmonary tuberculosis (EPTB). According to the global tuberculosis report of 2018, 10 million people fell ill with TB and there were an estimated 1.3 million TB deaths in 2017 worldwide [2]. In regard to global burden of disease, TB is predicted to maintain its status up to 2020 [3], despite international attention paid to the disease and effective prevention and control programs. The WHO has developed the End Tuberculosis Strategy, with a goal of 90% reduction in incidence and 95% reduction in mortality by 2035 [4]. In Tunisia, TB is of a public health concern. In 2014, there were 3170 new TB cases of any form, among which 59% were EPTB cases [5]. The notification rate of TB, all forms combined, was 28.8/ 100,000 population and the mortality rate was 2/100,000 population in the same year [5,6]. National Tuberculosis Control Program was implemented in Tunisia since 1959, which primary aim was to significantly reduce the burden of TB in compliance with the Sustainable Development Goals, which primary aim was to end TB related deaths, transmission and catastrophic costs by 2030 [7]. The highest rates were recorded in Southern Tunisia, where tuberculosis management is still challenging. Evidently, apart from economic losses, TB burden had an adverse impact either on patients’ quality of life or on their family members. Description of the epidemiological profile of the disease over time and characterizing its chronological trends could play an important role to assess the performance of control strategies for different periods, health development indicators and health planning. Since TB does not homogeneously affect the population, selected high-risk groups and studying their current and future trends should be identified in all settings as they deserve special attention and should be addressed specifically with additional interventions. Moreover, it is a useful tool for health-care professionals and decision-makers to improve planning policies attributed to TB and prioritization as well as the efficacy and efficiency of health program planning. In light of the challenging epidemiological situation and the lack of reliable and recent TB data, we sought to give an update about the current chronological trends of TB from 1995 to 2016 and to predict future trajectories of the TB epidemic up to 2030 in South of Tunisia.

## Materials and methods

### Study design

We conducted an ecological study of all notified TB cases from January, 1^st^ 1995 to December, 31^st^, 2016 in Southern Tunisia. This region hosted a high proportion of TB patients among the Tunisian population. All TB cases were notified by the Center of Tuberculosis Control, coordinated by the National Tuberculosis Surveillance Program. We collected data from the regional registries of TB, which received notified cases from both private and public health institutions.

### Population study and data collection

We included new TB cases of any form and any age notified during the study period. At enrollment, relapsing patients were excluded in order to calculate the incidence rates. Relapse of TB was defined as re-emergence of clinical symptoms in therapy-compliant patients after stopping anti-TB treatment, while this treatment appeared effective initially [8]. We excluded all cases with missing data, which represented less than 3% in our study. During the study period, the definition of the disease and the decision criteria of diagnosis were unified within the different medical centers of Southern Tunisia. All confirmed incident cases of TB were considered in our analysis. The diagnosis of TB was made by expert physicians who confirmed the diagnosis, established the anti-tuberculous treatment and addressed the notified case to the Center of Tuberculosis Control for additional medical care. In Tunisia, the diagnosis was done following the national TB guidelines, which were consistent with the updated WHO diagnostic criteria. [8]. It was based on bacteriological (sputum smear, urine) and/or histological proof. In default, it was based on strong clinical and radiological evidence followed by an adequate response to antitubercular treatment. The database variables included patients’ socio-demographic characteristics, the full address of TB patients and the TB form. We also collected information dealing with the treatment regimen (Fixed dose combination /separate tablets, therapy duration) and the disease outcomes (relapse, death, treatment failure).

### Tuberculosis incidence rates

According to the Tunisian Statistical Population Census, eligible patients were divided into 3 age categories: under 15 years, between 15 and 59 years and 60 years and above. We computed the yearly rate of tuberculosis incidence by dividing the total incident TB cases by the average population census calculated as follow: (Population in 2004 + Population in 2014)/2 based on the Tunisian National Institute of Statistics data. The incidence rate was calculated in the three age groups and in both genders and was expressed as the number per 100,000 population/year [9,10].

### Statistical analysis

Statistical analysis was performed using SPSS.20 software. The Kolmogorov–Smirnov test was used to assess the distribution of continuous variables. The results of continuous variables were presented as means ± standard deviation (SD) or medians and interquartile range (IQR). Those of categorial variables were presented as numbers and percentages. We used the t-test for independent samples to compare two means. For categorical variables, we used the Chi-square test in independent samples.

In order to analyze current chronological trends of TB incidence rates, the Joinpoint Regression Analysis program, version 4.5.1.0 was performed. This software is one of the most applicable in piecewise regression which is used for estimation of regression variables and drawing diagrams of fitted regression lines. Joinpoint fits a linear regression model to the data to detect periods with statistically distinct log-linear trends over time. This analysis identifies inflexion points (‘joinpoints’), using a series of permutation tests, with Bonferroni adjustment for multiple comparisons. A significance level of 0.05 was used for the permutation test, which determines the minimum number of “Joinpoints” necessary to fit the data. The use of a natural log-linear model enables the analysis of a constant percentage change in rate over time. The annual percent change (APC) within each segment was calculated with 95% confidence intervals (95% CI). Variation in trends of TB incidence for different age groups and for both genders was assessed over time.

To perform incidence projection for 2030, a period-cohort model was performed assuming a Poisson regression model for the count of the cases. We estimated the mean number of new cases of TB in each year, with lower and upper credible intervals (LCrI, UCrI). Then we calculated the estimated incidence rates up to 2030 based on the Tunisian Projected Population of 2021 [11]. A p value <0.05 was considered to be significant.

### Ethics statement

According to the Tunisian legislation, studies using patients’ records used in our retrospective study do not require ethical approval. All data were fully anonymized before we accessed them and no individual person can be identified.

## Results

### Patients’ characteristics

The total number of new notified cases over the 22-year study period was 2771, all forms combined. At enrollment, the median age of patients was 38 years (IQR = [25–55 years]). A total of 1508 (54.4%) patients were males, with a sex ratio (Male/Female) of 1.19. There were 2068 (74.6%) patients aged between 15 and 59 years. We recorded 1650 (59.5%) cases of EPTB, among whom the main EPTB forms were lymph node in 754 (45.7%) cases, followed by pleural TB in 232 (14%) cases, urogenital in 188 (11.4%) cases, abdominal in 182 (11%) cases, bone and joints in 121 (7.3%) cases, neuro-meningeal in 66 (4%) cases and cutaneous TB in 44 (2.6%) cases. Multifocal tuberculosis, defined as 2 different anatomical sites at least involved accounted for 2.4% (N = 66 cases). Table 1 shows a comparison of basic demographic and clinical characteristics between pulmonary and extra-pulmonary tuberculosis patients at baseline. We found that PTB was significantly more common in males (p<0.001). According to residence, PTB was significantly more frequent in rural districts (p = 0.009). The treatment regimen was based on fixed-dose combination in 26.4% of cases. The therapy duration was statistically longer in EPTB patients (p<0.001). The case fatality rate was significantly higher in PTB (p = 0.035).

**Table 1:** Comparison of basic demographic and clinical characteristics between pulmonary and extra-pulmonary tuberculosis patients.

Variables	Total (N = 2771)	PTB (N = 1121)	EPTB (N = 1650)	p-value
**Gender (N, %)**				
Males	1508 (54.4)	759 (67.7)	749 (45.4)	***<0*.*001***^μ^*
Females	1263 (45.6)	362 (32.3)	901 (54.6)	
**Age categories (years) (N, %)**				
0–14	167 (6)	30 (2.7)	137 (8.3)	***<0*.*001*** ^μ^*
15–59	2068 (74.6)	861 (76.8)	1207 (73.2)	***0*.*035***^μ^*
≥60 years	536 (19.4)	230 (20.5)	306 (18.5)	0.190 ^μ^*
**Residence** (N, %)				
Rural	1144 (41.3)	496 (44.2)	648 (39.3)	***0*.*009*** ^μ^*
Urban	1627 (58.7)	625 (55.8)	1002 (60.7)	
**Treatment regimen**				
Fixed dose combination (N, %)	732 (26.4)	317 (28.3)	415 (25.2)	0.067 ^μ^*
Separate tablets	2039 (73.6)	804 (71.7)	1235 (74.8)	
**Therapy duration (months) (Mean ± SD)**	7.46 ± 4.3	6.6 ± 3.5	8 ± 4.6	***<0*.*001*** ^μ^**
**Disease outcomes (N, %)**				
Relapse	10 (0.4)	3 (0.3)	7 (0.4)	0.700 ^μ^*
Death	79 (2.9)	41 (3.7)	38 (2.3)	***0*.*035*** ^μ^*
Treatment failure	5 (0.2)	4 (0.4)	1 (0.1)	0.100 ^μ^*

N: Number; PTB: Pulmonary tuberculosis; EPTB: Extra-pulmonary tuberculosis; SD: Standard deviation

*: Chi-square test

**: t-test.

^***μ*:**^ p-value between PTB and EPTB.

### Tuberculosis incidence and mortality rates stratified by gender and age group

Over the 22-year-study-period, the mean annual number of all TB forms was 125.95 cases/year and the annual incidence rate was 13.91/100,000 population/year. The mean incidence rates were 5.63 and 8.28/100,000 population/year for PTB and EPTB, respectively. Overall, the mortality rate of TB was 0.39/100,000 population/year.

When stratified by age group, the incidence rate as well as the mortality rate of TB were significantly higher in patients aged 60 years and above than the other age groups, all forms combined. Sex-specific incidence rates showed significantly higher rate of PTB for males (7.54/100,000 population/year vs 3.67/100,000 population/year; p = 0.0018) and significantly higher rate of EPTB for females (9.14 /100,000 population/year vs 7.44/100,000 population/year; p<0.001). Mortality rates of PTB were significantly higher in males (p = 0.009) (Table 2).

**Table 2:** Annual number of cases, incidence and mortality rates of pulmonary and extra-pulmonary tuberculosis stratified by gender and age groups.

Variables	Average population	N of cases over 22 years (mean annual number)	Incidence rate /100 000 population/year	Number of deaths	Mortality rate /100 000 population/year
**Overall tuberculosis cases**					
**Total**	905339	2771 (125.9)	13.91	79	0.39
**Age groups**					
<15 years	226162	167 (7.6)	3.36	1	0.02
[15–59 [years	582813	2068 (94.0)	16.12	34	0.26
≥60 years	96317	536 (24.3)	25.22	44	2.07
p-value*			*<0*.001**		*<0*.001**
**Gender**					
Male	457485	1508 (68.5)	14.98	53	0.52
Female	447853	1263 (57.4)	12.81	26	0.26
p-value			***<0*.*001***		***0*.*003***
**Extra-pulmonary tuberculosis**					
**Total**	905339	1650 (75.0)	8.28	38	0.19
**Age groups**					
< 15 years	226162	137 (6.2)	2.75	1	0.02
[15–59 [years	582813	1207 (54.9)	9.41	17	0.13
≥ 60 years	96317	306 (13.9)	14.43	20	0.94
p-value*			*<0*.001**		*<0*.001**
**Gender**					
Male	457485	749 (34.0)	7.44	24	0.24
Female	447853	901 (40.9)	9.14	14	0.14
P value			***<0*.*001***		0.12
**Pulmonary tuberculosis**					
**Total**	905339	1121 (51.0)	5.63	41	0.20
**Age groups**					
<15 years	226162	30 (1.4)	0.29	0	0
[15+59 [years	582813	861 (39.1)	6.71	17	0.13
≥60 years	96317	230 (10.4)	10.85	24	1.13
p-value*			*<0*.001**		***<0*.*001***^***μ***^
**Gender**					
Male	457485	759 (34.5)	7.54	29	0.29
Female	447853	362 (16.4)	3.67	12	0.12
p-value			***0*.*0018***		***0*.*009***

N: Number, p-value: Using Chi-square test

*: Global p-value of the 3 age categories.

**: p-value <0.05 in each pair comparison of age groups.

^μ:^ p-value between 15–59 and ≥60 years age groups.

### Current chronological trends of tuberculosis by gender, age groups, residence and main form

**Fig 1:**
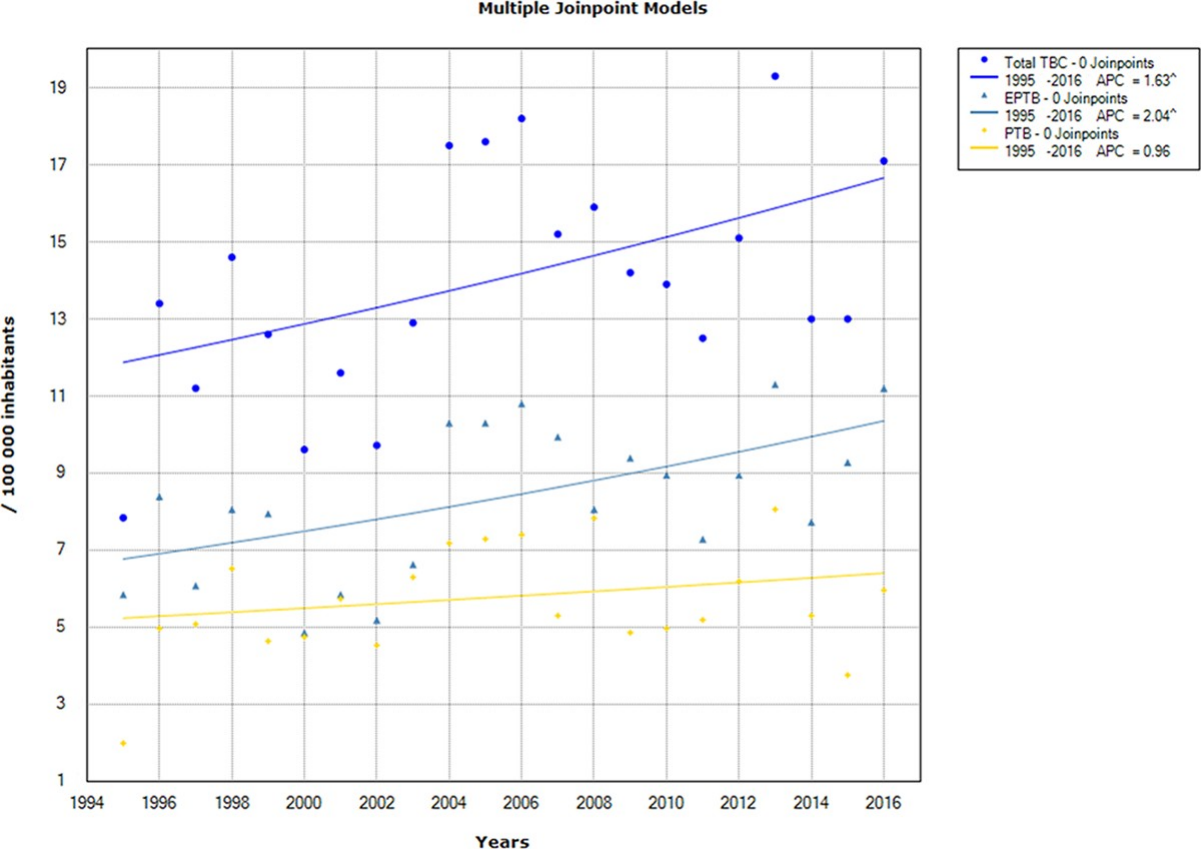
Chronological trends analysis of overall tuberculosis (TB), pulmonary tuberculosis (PTB) and extra-pulmonary tuberculosis (EPTB) incidence rates between 1995 and 2016.

On analysis of different TB subgroups trends using Joinpoint regression, there was a significant increase in the TB incidence rate, all forms combined, from 7.84/100,000 population in 1995 to 17.60/100,000 population in 2016, with an APC of 1.63% (95% CI: [0.2–3]). Likewise, EPTB had a significant growing trend, with an incidence rate rising from 5.85/100,000 population in 1995 to 11.20/100,000 population in 2016, with an APC of 2.04% (95% CI: [0.6–3.5]). As to PTB, the incidence rate increased from 2/100,000 population in 1995 to 5.96/100,000 population in 2016, but with no significant change over time (APC = 0.96%; 95% CI: [-0.9–2.8]) (Fig 1).

**Fig 2:**
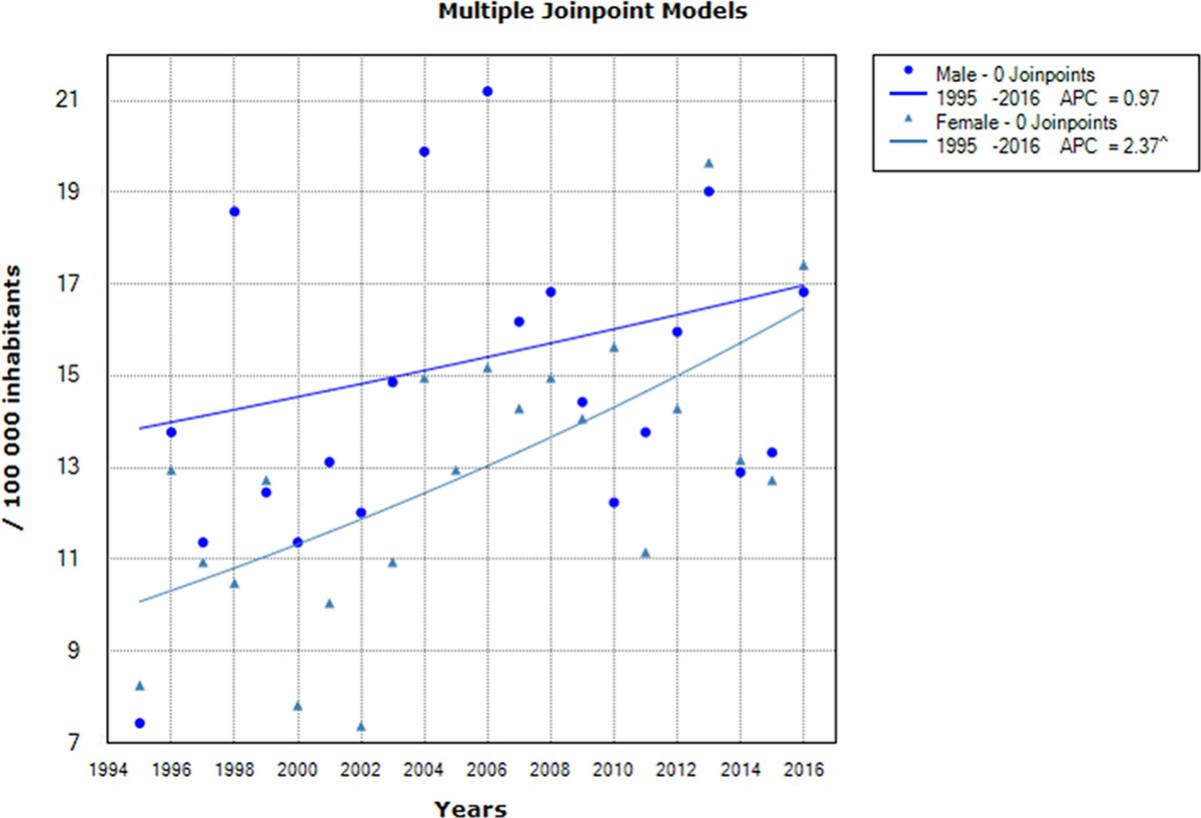
Chronological trends analysis of tuberculosis incidence rates by gender between 1995 and 2016.

In regard to trends analysis of TB incidence by age groups, children aged 0–14 years old showed a significant increase between 1995 and 2016 (APC = 4.48% 95 CI% [1–8.1]). The age groups 15–59 and 60 years old and above had an increasing trend, but the incidence rate variation trend was insignificant at level of 95% (Fig 2).

**Fig 3:**
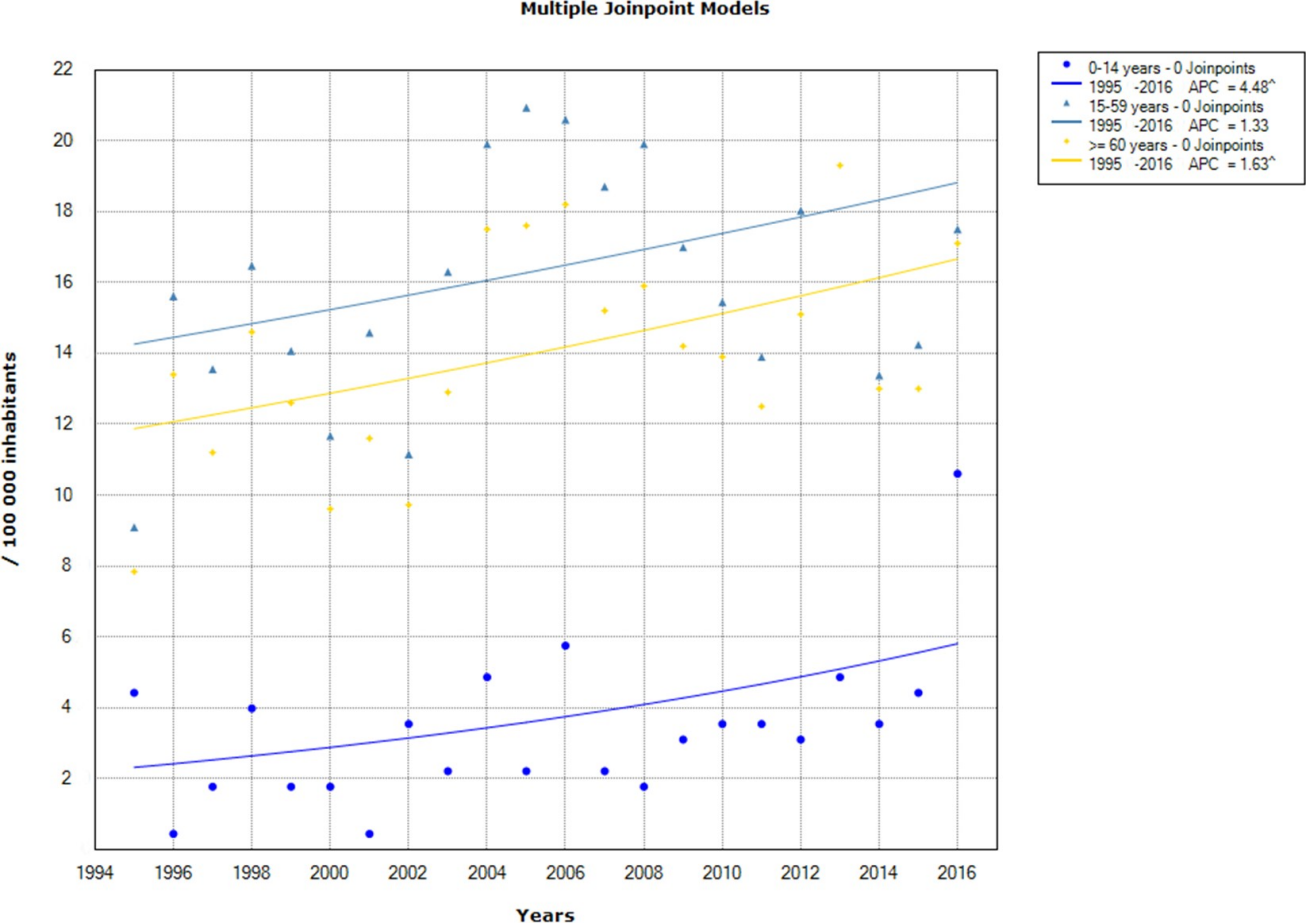
Chronological trends analysis of tuberculosis incidence rates by age groups between 1995 and 2016.

Sex-specific incidence rates showed a significant increase in TB incidence for females, from 8.26/100,000 in 1995 to 17.42/100,000 population in 2016, with an APC of 2.37% (95% CI [1–3.7]), while no significant change was noted in males (Fig 3).

**Fig 4:**
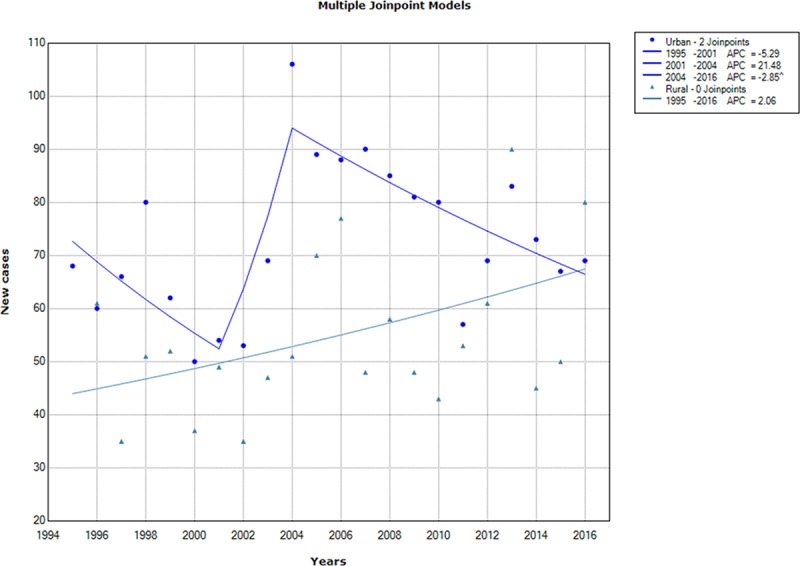
Chronological trends analysis of tuberculosis annual number in urban and rural districts between 1995 and 2016.

According to residence, trends analysis of the reported annual number of TB in urban districts showed 2 breakpoints (2001 and 2004), then a significant decrease was recorded between 2004 and 2016, with an APC of -2.85% (95% CI = [-4.8; -0.9]). Otherwise, the increase of TB number in rural districts was insignificant (Fig 4).

**Fig 5:**
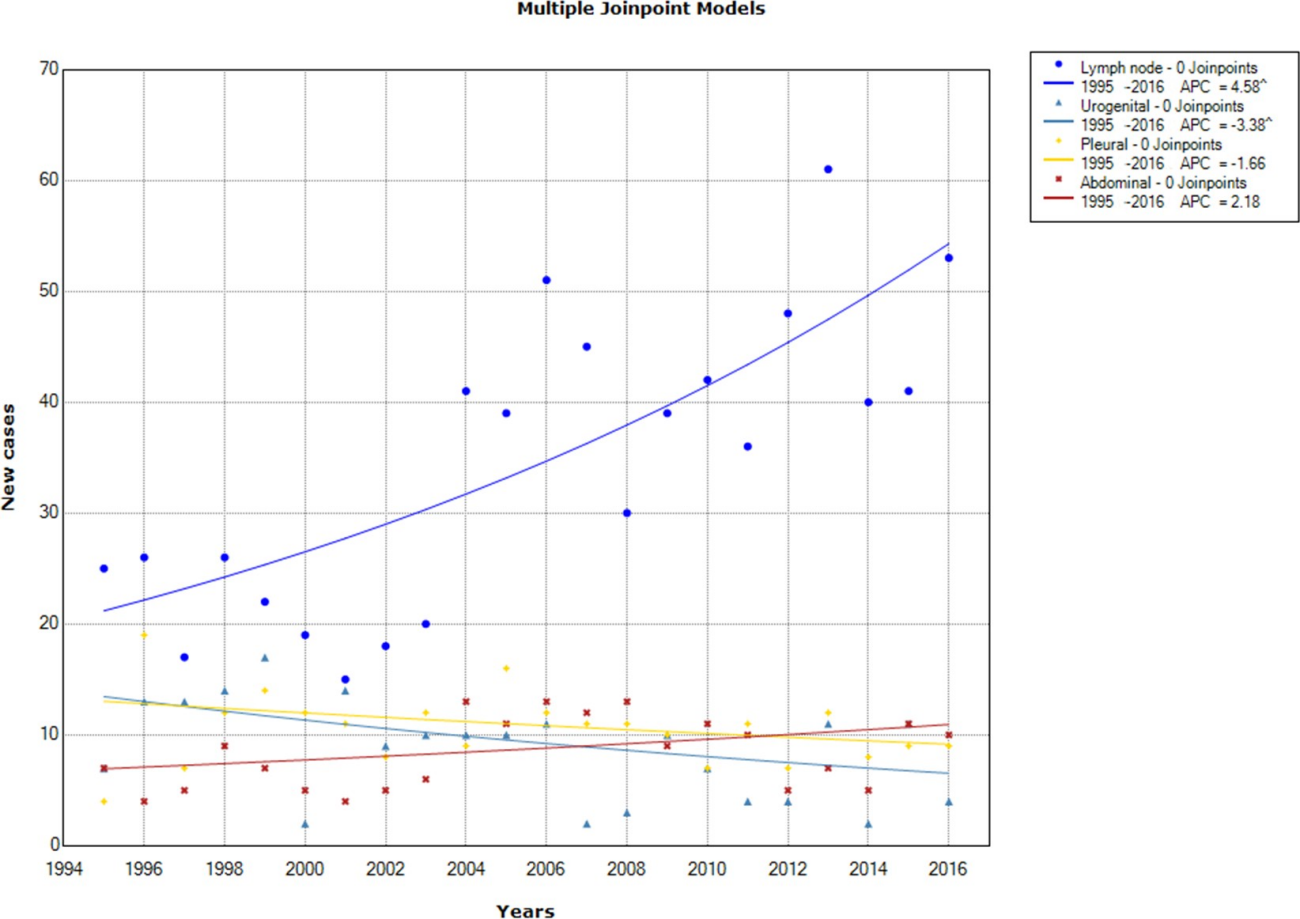
Chronological trends analysis of the extra-pulmonary tuberculosis annual number by form between 1995 and 2016.

Trends analysis of annual EPTB new cases stratified by TB forms showed a significant increase in lymph node tuberculosis (APC = 4.58%; 95 CI% = [2.7–6.5]) between 1995 and 2016, but a significant decline in urogenital tuberculosis (APC = -3.38%; 95% CI [-6.4; -0.3]). Abdominal and pleural tuberculosis showed no significant variation over time (Fig 5).

### Projected incidence rates of tuberculosis up to 2030

**Fig 6:**
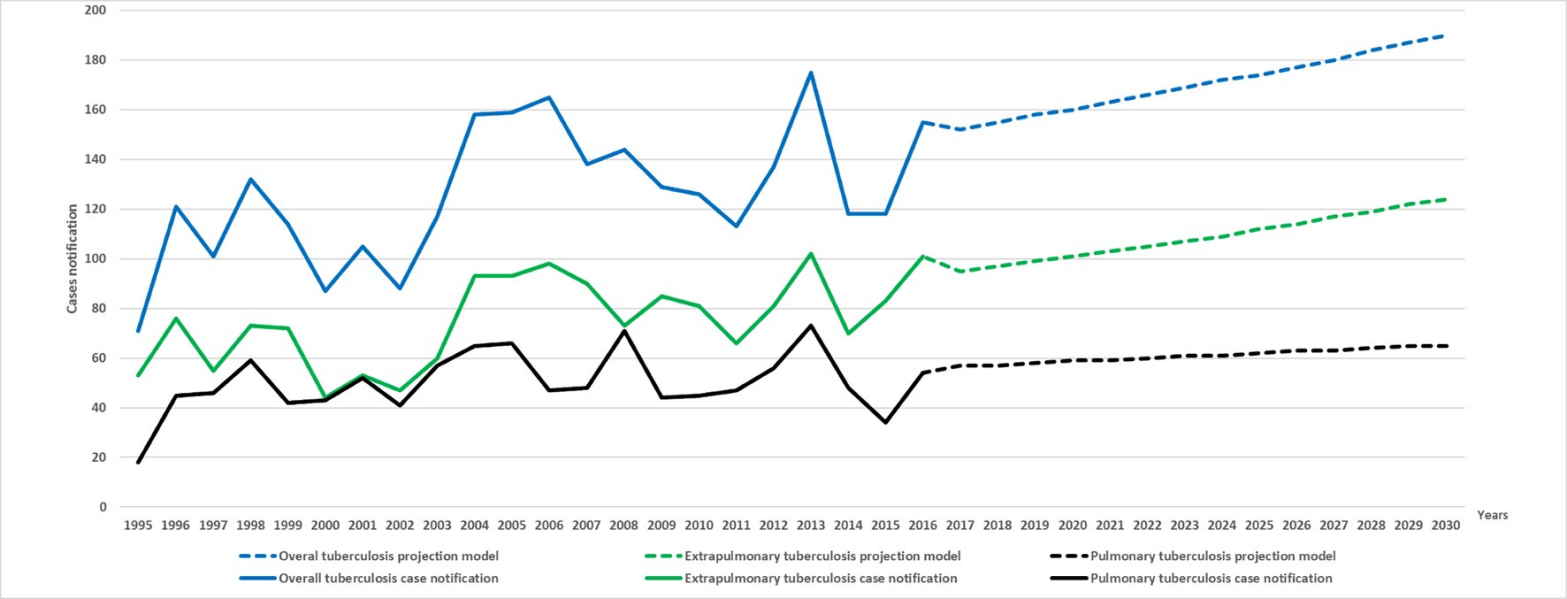
Annual number new cases for the observed (1995–2016) and predicted (2017–2030) periods of total, pulmonary and extra-pulmonary tuberculosis.

Forecasting into the future using the past epidemiological data showed that the extrapolated future values of TB incidence over the period 2017–2030 may continue to increase. We expect that the annual number of TB new cases would be more than 190 [LCrI = 164, UCrI = 220] for total TB and 124 [LCrI = 103, UCrI = 150] for EPTB by 2030. Thus, the estimated incidence rates would increase to 18.13/100,000 population and 11.80/100,000 population, respectively. With regard to PTB, projected yearly incidence showed no substantial variation over the coming decade: the predicted number of new cases would attend 65 [LCrI = 52, UCrI = 82] by 2030, with an estimated incidence rate of 6.20/100,000 population (Fig 6).

## Discussion

Using TB surveillance data of 2771 patients, we conducted the first assessment of TB burden in South of Tunisia, overall and within subpopulations of different socio-demographic characteristics in the last two decades. This study reported that all TB forms incidence rate as well as EPTB had significantly increased in the last two decades and will continue to rise in the next 10 years. This alarming upward trend was illustrated particularly among high-risk groups, including children, females, and rural districts residents.

Our analysis described the distribution of the TB burden by form in Southern Tunisia. EPTB was predominant (59.6%). This alarming finding reflected deficiency in EPTB control programs and inadequate preventive strategies in this region. This high prevalence could be due to the spreading of HIV infection and TB drug resistance that disrupted the TB control, resulting in a reactivation of *Mycobacterium*. Previous studies reported different rates of EPTB, such as 35.3% in east-central Tunisia [12], 57.5% in Riyadh, Saudi Arabia [13], 48.2% in Western Nepal [14], 36.1% in Cameroon [15] and 17% in 8 regions in Morocco [16]. A similar study done in India reported that EPTB was more common in female, notably lymph node TB, central nervous system TB and TB of bones and joints [17]. In fact, predilection to involve one site over the other depends on the host factors as well as demographic and social factors contributing to the development of EPTB [17,18]. Besides, the majority of TB cases (74.6%) belonged to the 15–59 years age group, who are young and working individuals, highlighting the socio-economic burden of TB. These findings were consistent with similar results of higher frequency of TB in younger individuals reported by other studies as well [17,19,20].

Tunisia is at intermediate endemicity for TB with a reported incidence ranging from 23/100,000 population in 2005 to 31/100,000 population in 2012 [21]. Our study showed slightly lower incidence during the study period than the national rate, reflecting regional disparities in health-care facilities and in efficacy of National Tuberculosis Program. A study conducted in Iran showed that the mean incidence rates were 8.34, 5.25 and 7.6/100,000 population/year for all TB forms, PTB and EPTB, respectively, which were consistent with our results. In our study, the highest incidence rate of TB was recorded among elderly. The same finding was exhibited in a previous study, with a TB incidence rate exceeding 40/100,000 population/year between 2010 and 2012 [22]. Eastern Mediterranean and South East Asia TB notifications have been increasingly evident in older people, peaking amongst those aged ≥65 years old [21]. Possible explanation for that is the fact that elderly people have an increased susceptibility to infectious diseases, particularly of the respiratory tract [23]. Therefore, future epidemiological surveillance and development of diagnostic tests and novel treatment regimens should all include a focus on ageing people.

According to the WHO, the TB incidence rate is falling at about 2% per year between 2000 and 2017 worldwide [2]. In Tunisia, a middle-income country of 11 million population, TB burden has been steadily declining since the implementation of a national TB program in 1959. Otherwise, in the last two decades, Tunisia, like many other countries, experienced a substantial increase in EPTB cases, reaching 68% of all new TB cases registered in 2017 [24]. Our study highlighted a significant increase in all TB forms incidence rate, notably in EPTB between 1995 and 2016. An important aspect must be taken into consideration: trends of TB incidence over time should be interpreted according to the socio-economic status of each region. In South East of Nigeria, the number of all TB cases reported annually showed a rising trend and the number of reported EPTB cases increased markedly between 2000 and 2008 [25]. These trends were also observed in India, with a sustained increase in the number of EPTB cases diagnosed and treated [17]. Similarly, a study conducted in Iran pointed to an increase in TB incidence, but with no significant change between 2005 and 2015 [22]. In sub-Saharan Africa, the average trend was upwards between 1997 and 2006 (average APC 1.8%) [26]. On the other hand, TB incidence was declining more quickly in countries that had a higher human development index, lower child mortality and access to improved sanitation [26]. Likewise, the number of TB and incidence rates declined significantly in the USA, the UK and in Argentine between 2000 and 2011 [27,28] and did not increase in Serbia between 1990 and 2004 [29]. This decrease was likely related to the increase in vaccination coverage and the decrease in children mortality rate [30].

In children aged under 15 years, previous studies reported a significant decrease in TB incidence between 1995 and 2012 in Iran [31] and between 2011 and 2015 in Uganda [32], which was contrasting with our study. In 2014 and according to figures from the Primary Health Care Directorate in Tunisia, the incidence of TB in the age group 0–14 years was 7.34/100,000 population for boys and 8.61/100,000 population for girls [33]. TB in young children differs from adults in that the risk of rapid progression to active TB is higher in children than in adults, and then higher morbidity and mortality rates in middle and low-income countries [34]. Therefore, investment in improving children health and health services should be as important as targeted strategies for controlling TB.

The study’s findings were in line with the belief that TB is a poverty-related disease and that its burden lies more with rural districts [35], where a substantial rise in TB annual number was observed, as opposed to urban districts showing a significant decline over time. Possible explanations for this trend are deficiency in primary health care, lack of access of sick persons to healthcare services, poor housing and environmental conditions, food insecurity, financial difficulties, illiteracy and unfavorable psycho-social circumstances in poor regions, which have been reported as major determinants of TB transmission [36].

The spectrum of EPTB in our region has significantly changed within the last two decades, with a significant rise in lymph node TB incidence. In Tunisia it was estimated that 50% of all EPTB cases affected lymph node, mainly caused by *Mycobacterium bovis* [37,38]. This situation prevails currently in most countries where bovine TB is endemic, with no effective control measures. High rates of raw milk consumption and close contact with animals, markedly in rural areas in our region may argue our findings. This argument could also explain in part the rise of EPTB incidence in women, who were mostly farmer workers, and consequently were more exposed to lymph node TB. Other studies conducted in India and Russia reported an increase in bone and joint, neuro-meningeal and other forms of EPTB [17,39].

Projected incidence rates of TB in our region predicted alarming rates up to 2030, notably in EPTB. These results were contrasting with previous studies showing the projected scenario of TB over the coming decade in developed countries, where TB incidence will decline by 1.3% per year in New York [40] and will continue to fall by 42% by 2030 in Quebec [41]. Thus, there may be a need for the TB program to consider incorporating effective treatment regimens in highly endemic region, notably in South of Tunisia, to revise public health measures and to improve life standards in this region in order to reduce the TB burden.

Our study provided accurate and exhaustive data including a large number of TB incident cases over the last two decades and the projected trajectory up to 2030. These estimates provided useful information on the TB disease burden for public health officials to devise interventions to halt and reverse the trajectory. However, our study had some limitations. Firstly, because of its retrospective nature, the patients were not followed-up during the study period and the treatment outcome as well as the response to therapy could not be assessed. Changes in trend of some indices such as treatment success rate, case detection and mortality rate were not assessed. Secondly, deficiencies in the databases included possible missing or incomplete data, and potential biases and errors during date entry. Besides, the under reporting of the notified cases because of passive surveillance system for TB control program may under-estimate the real incidence rate of TB. Another limitation of the study is failure to examine the effect of related factors on the TB incidence such as HIV co‑infection, clinical signs and individual’s socioeconomic status. Additional nation-wide and prospective studies are needed to better understand the characteristics of such patients.

## Conclusion

Our study provided an insight into the magnitude of TB in Southern Tunisia. TB incidence was higher among the productive age group. Significant increase in the trend of TB globally and among high-risk groups, notably children, females, and rural districts residents in the last two decades and up to the next one, is a point of concern highlighting the importance of strengthening health care services towards these groups. Lymph node TB was epidemic in our region, suggesting the urgent need to establish preventive strategies for controlling zoonotic tuberculosis transmission. These findings provided useful measurements to health-decision makers to better evaluate the efficiency of TB program in our country and to prioritize curative and preventive interventions accordingly.

## Supporting information

S1 File Repository data of the manuscript. (CSV)
